# Over the Edge: Extending the duration of a reconsolidation intervention for spider fear

**DOI:** 10.1038/s41398-022-02020-x

**Published:** 2022-06-23

**Authors:** Anna I. Filmer, Jacqueline Peters, Lara A. Bridge, Renée M. Visser, Merel Kindt

**Affiliations:** Department of Clinical Psychology, University of Amsterdam, Amsterdam, Netherlands

**Keywords:** Psychology, Learning and memory

## Abstract

Pharmacologically disrupting fear memory reconsolidation dramatically reduces fear behaviour. For example, 2–3 min of tarantula exposure followed by 40 mg of propranolol HCl (i.e., a reconsolidation intervention) abruptly decreased spider avoidance, an effect that persisted one year later. However, the success of reconsolidation interventions is not guaranteed: Pavlovian fear-conditioning research shows that the window to target memory reconsolidation is small and easy to miss. If exposure is too long to trigger reconsolidation, but too short for extinction learning, an inactive transitional limbo state occurs, rendering the fear memory unchanged and insensitive to amnesic agents. In this pre-registered study, we aimed to find this behaviourally-controlled boundary condition. Spider-fearful participants underwent a ~3 min (*n* = 23) or ~14 min (*n* = 20) exposure to a tarantula, intended to trigger reconsolidation or the limbo state respectively, followed by 40 mg of propranolol. We expected greater spider fear reduction after 3 than 14 min of exposure. Unexpectedly, there were no group differences on any outcome measures. In both groups, Bayesian analysis revealed a marked reduction in fear behaviour towards a generalisation stimulus (a house spider) accompanied by lower self-reported distress, with a sharp decline in spider fear scores two days after treatment that persisted one year later. Possible explanations include that the boundary conditions of reconsolidation are wider in older and stronger memories than experimentally-induced fears, or that alternative processes caused the treatment effects. Although the mechanism is unclear, these results carry a tentative promising message for the potential of brief reconsolidation-targeting interventions to mitigate irrational fears.

## Introduction

Fear is highly adaptive: it guides our behaviour, protecting us from danger. However, the associative networks thought to underlie our learned fear responses (i.e., fear memories) are resistant to decay, and can affect our behaviour after their adaptive purpose has passed, contributing to anxiety disorders [1]. When fear memories are reactivated, they can undergo an updating process called reconsolidation. Here they become vulnerable to certain drugs such as anisomycin or propranolol (i.e., amnesic agents), which abruptly reduce fear behaviour [2]. There has been much interest in harnessing the disruption of reconsolidation of fear memories for therapeutic benefits (i.e., “reconsolidation interventions” — note that we use this term for treatments that are intended to disrupt reconsolidation, not to indicate that we know that the mechanism is reconsolidation). The appeal is clear: with a single memory reactivation followed by the one-time administration of an amnesic agent they offer the opportunity to target maladaptive memories, promising lower rates of relapse [3]. Research on fears acquired in the laboratory (i.e., conditioned fears) indicates that reconsolidation depends upon the learning history, including the age and strength of the targeted memory, where older and stronger memories are more resistant to reconsolidation [4, 5], and the interaction between what has already been learned and what occurs during the reactivation procedure (e.g., refs. [6, 7]). For clarity, throughout this article, we use the term reactivation procedure to denote memory retrieval procedures where the subsequent occurrence of reconsolidation is uncertain. In the laboratory, these critical factors can be closely controlled by varying the conditioning or reactivation procedures. Yet even working with such simple associations, there have been failures to replicate the reconsolidation effect [8–10]. Almost all previous studies on clinical and pre-clinical reconsolidation interventions have used study-specific reactivation procedures combined with an amnesic agent or a placebo to determine whether that particular reactivation procedure triggered reconsolidation [11–14]. The inconsistency in the results is unsurprising given the substantial variation in the procedures employed (e.g., Elsey & Kindt, 2021 [15]; see Walsh et al. [16] for a review), and that the window to target the process of memory reconsolidation seems to be small and easy to miss [17]. We currently do not have a validated, non-invasive, independent marker that can be used during reactivation procedures to indicate whether reconsolidation is triggered. Therefore, the current study aimed to find a behaviourally-controlled boundary condition of reconsolidation in a naturalistic fear, to inform the development of future reconsolidation interventions. Specifically, we focussed on the duration of the reactivation procedure.

The phenomenon of memory reconsolidation has been most convincingly demonstrated in Pavlovian fear-conditioning paradigms, where an innately aversive experience (i.e., unconditioned stimulus; US), such as pain, becomes associated with a neutral or ambiguous stimulus (i.e., conditioned stimulus; CS), such as a tone [2]. Encountering the CS activates the association with the US, resulting in fear of the CS. When testing for reconsolidation, researchers typically use a reactivation procedure, which usually involves physically or mentally exposing the participant to the CS in some way. To assess whether the reactivation procedure actually triggered reconsolidation, researchers then apply an amnesic agent with the intention of blocking this reconsolidation. A continued fear response is taken as an indication that the reactivation procedure on the previous day did not trigger reconsolidation, the amnesic drug was not effective, or both. Contrastingly, a dramatic reduction in the fear response is taken as an indication that the reactivation procedure successfully triggered reconsolidation, which was subsequently disrupted by the amnesic agent.

Such fear-conditioning paradigms have shown that prediction error is critical in triggering reconsolidation. Prediction error occurs if the reactivation procedure contains new information about the CS–US association (i.e., match/mismatch between the expected and actual outcomes); for example, the unpredicted presence or absence [6], timing [18], or intensity [19, 20] of the US following exposure to the CS. Note that we use the term prediction error in a slightly different way from the fear-conditioning literature, where it is generally defined as fixed instances of outcome—expected outcome. While it is not possible to directly operationalise this in clinical science, as a proxy for the laboratory definition of prediction error we use the same term both to link it with associative learning theory and to be consistent with other translational research. However, as prediction error cannot be as precisely quantified in this context, we can only use less specific or relative terms (e.g., “more”, “less”, “amount”). Pavlovian fear-conditioning research in animals and humans has indicated that while prediction error is a necessary condition for reconsolidation, it is not sufficient. Instead, the amount of prediction error in the reactivation procedure is critical to which post-retrieval memory process occurs [21]: Reconsolidation occurs when prediction error is present but limited, extinction learning occurs when prediction error is extensive, and limbo—an insensitive state in between reconsolidation and extinction—occurs when the amount of prediction error is too high for reconsolidation and insufficient for extinction [5, 7, 22]. Note that, prediction error (or the quantity thereof) is not an independent quality of the reactivation procedure: it depends entirely upon how the experience at the reactivation procedure aligns with the learning history. This makes it a real challenge to identify absolute criteria for triggering reconsolidation in clinical practice, where the learning history is unknown.

While we do not have absolute criteria to determine whether a reactivation procedure triggers reconsolidation, limbo, or extinction, animal research has found a neurobiological marker that distinguishes between the processes (calcineurin levels in the basolateral amygdala [22]). Both extinction and reconsolidation require *de novo* protein synthesis and can therefore be disrupted by concomitantly applying pharmacological agents that directly or indirectly inhibit this (e.g., anisomycin, propranolol HCl resp.) [22, 23]. In extinction new emotionally neutral memory traces are formed and compete with the fear memory, thus disrupting extinction results in continued fear expression [24, 25]. In reconsolidation new information is incorporated into the existing memory trace [2, 22], therefore disruption achieves the opposite effect, reducing the emotional valence and behavioural expression of the existing fear memory after sleep [2, 14, 26]. Such pharmacological agents do not affect subsequent fear-memory expression when applied during the limbo-state [22], the phase between reconsolidation and extinction. In humans, we cannot directly observe neurobiological markers to inform us about which phase we are in, and in clinical practice we do not know the learning history (which varies dramatically for every patient), making careful control of the interplay with the reactivation procedure untenable. We must currently rely on the outcome of the intervention following sleep, after the effects of a pharmacological manipulation are clear [26], to retrospectively determine which process the reactivation procedure triggered. To facilitate effective and efficient reconsolidation interventions, we require methods to behaviourally control prediction error, regardless of learning history, that we can use to determine which process we trigger in clinical practice.

In the laboratory the amount of prediction error in a reactivation procedure has been manipulated in various ways, including the following; (1) increasing the number of prediction error events that occur (e.g., CS-alone presentations) [5, 7, 22, 27], and (2) increasing the duration of unreinforced exposure to the CS after the prediction error event occurs (e.g., continuing exposure to the CS after the absence of reinforcement has become apparent) [28]. Note that the operationalised manipulation in both is a longer exposure to the unreinforced CS: (1) is longer because it takes more time to include more prediction error events, and (2) is longer because there is a single larger prediction error event. Such laboratory studies have provided key insights into the boundary conditions of reconsolidation. In a recent study [5], following Pavlovian fear conditioning, crabs underwent reactivation procedures with varying numbers of prediction errors (i.e., CS-alone presentations), followed by an amnesic agent. When the conditioning was brief, a small number of CS-alone presentations triggered reconsolidation, while a medium number of CS-alone presentations caused limbo and a large number of CS-alone presentations led to extinction learning. However, when the original conditioning procedure was lengthened, both the small and medium number of CS-alone presentations triggered reconsolidation. This showed that the bounds of the number of prediction error events required at the reactivation procedure to trigger reconsolidation vary as a function of the learning history. The translation of such paradigms to clinical practice presents a key challenge, as here the precise nature of prediction error is hard to define or divide into fixed events [29]. Thus, in the absence of a clear-cut prediction error event, it may be that the duration of a reactivation procedure can serve as an experimental proxy for the amount of prediction error. This aligns with observations in humans, where reconsolidation and extinction of the relatively young and weak memories are triggered by seconds or minutes of reactivation procedures respectively [7]. Contrastingly, for the older and stronger memories targeted in clinical practice, reconsolidation interventions include a reactivation procedure of minutes (e.g., refs. [14, 30]), and exposure therapy, where the underlying memory process is thought to be extinction, typically lasts 2–3 h across single or multiple sessions (e.g., refs. [31, 32]). However, currently, the duration of reactivation procedures has only been directly shown to determine post-retrieval memory processes in fear-conditioning paradigms.

Previous research by Soeter and Kindt has found a successful reconsolidation intervention for spider fear, which provides an opportunity to further examine the boundary conditions of memory reconsolidation in naturalistic fears [14]. In this study, spider-fearful individuals underwent a reactivation procedure consisting of a single ~2 min interaction with a tarantula, followed by a placebo or propranolol. Those that received propranolol abruptly reduced their fear behaviour at later tests, long after the drug had left their systems. This occurred in a nearly binary fashion, where all those that followed the reactivation procedure with propranolol touched an adult tarantula four days later in a behavioural approach task, and reported less distress during this task. They also showed more approach towards a generalisation stimulus (a baby tarantula) 11 days after treatment. Self-declared spider fear measured by spider fear questionnaires, which had not reduced 11 days after treatment, decreased at the following measurement point three months after treatment, with no relapse one year later.

In the current pre-registered study, we modified this reactivation procedure such that it could be extended. Participants approached a tarantula and stayed close to it while an experimenter prompted the tarantula to move. For the extended reactivation procedure, we aimed to target the limbo state, selecting a time that was considerably longer than the 2 min interval used in the previous study, but much shorter than the 2–3 hr intervals generally used to trigger extinction in exposure therapies. In piloting 8- to 15 min reactivation procedure durations (*n* = 3; starting range determined based on MK’s clinical experience), after excluding a participant who would not have met the final study inclusion criteria, neither remaining participant touched the tarantula after treatment (our primary outcome variable). Contrastingly, for piloted individuals in the brief condition (*n* = 2), both participants showed much stronger spider behavioural approach after treatment (Tarantula Behavioural Approach Task step ≥ 7; see subsection Tarantula Behavioural Approach Task for more details). We selected the longer piloted duration for the extended reactivation procedure to ensure that the length was sufficiently different to the brief reactivation procedure. Therefore, in the current experiment, spider-fearful individuals underwent a reconsolidation intervention consisting of a brief (2- to 5 min) or extended (13- to 15 min) reactivation procedure with a tarantula followed by 40 mg of propranolol. We expected that reconsolidation would only occur and be subsequently disrupted following brief but not extended reactivation procedures, resulting in lower spider fear after treatment in the brief compared to the extended group. Specifically, we predicted higher levels of spider approach and lower levels of self-reported fear while approaching both the tarantula used in the treatment and a generalisation stimulus (a house spider) two days after treatment. Approach behaviour towards the tarantula was pre-registered as the primary dependent variable. In previous research, disrupting reconsolidation reduced self-declared spider-fear scores between the spider-approach tasks shortly after treatment and three months later [14]. However, because they were not measured between these time points, it is not clear when this reduction occurred. Therefore, we also assessed self-declared spider fear the day after the post-intervention approach tasks. The pre-registration did not include the 3-month or 1-year questionnaire follow-ups.

## Methods and materials

### Pre-registration

We pre-registered study procedures, exclusion criteria, the sequential analysis to determine the sample size, and confirmatory analyses on the Open Science Framework (https://osf.io/2t5nc/). After data collection started, we added a further stopping criterion due to the high exclusion rate (https://osf.io/fbjhw). This decision was made blind to the sequential analyses’ results.

### Participants

**Fig. 1:**
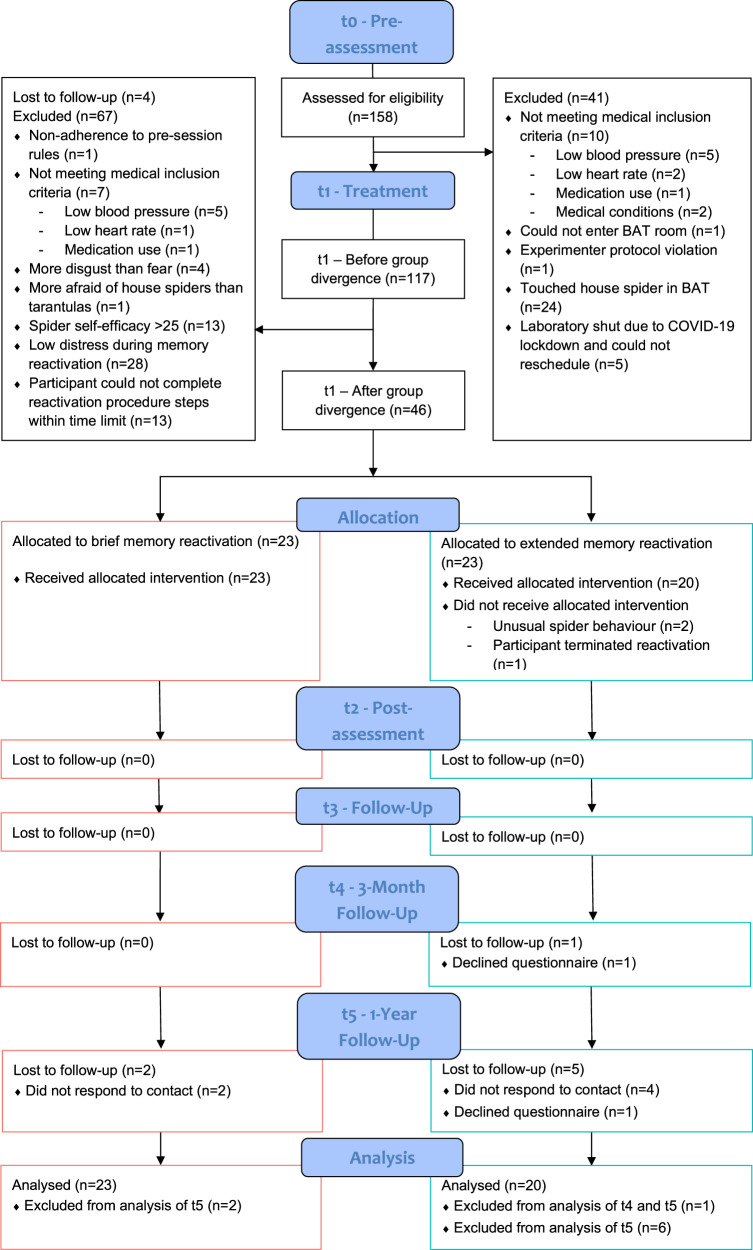
The participant flow and in-session exclusion criteria. Blood pressure was defined as too low when the systolic blood pressure was below 100 or the diastolic blood pressure was below 60. Heart rate was too low if it was below 60, or below 50 for those who did more than 7 h of exercise a week, unless the heart rate rose above the cut-off after 2 min of light exercise. For more specific details on all exclusion reasons see Supplement B. BAT Behavioural Approach Task.

We recruited clinically and sub-clinically spider phobic participants via the University of Amsterdam (UvA) recruitment portal, flyers, social media advertisements, and the UvA Psypoli. The reward was a small financial sum or study credits. As an effective reactivation procedure depends upon generating an expectation of threat [33], we pre-registered strict inclusion criteria to maximise the likelihood that our standardised reactivation procedures would be suitable. Notably, participants had to (1) report at least one distress score of 65 or higher in the first two ratings of the reactivation procedure, (2) indicate before the treatment that they were not more afraid of house spiders than tarantulas, as the treatment used a tarantula, and (3) not be more disgusted by than afraid of spiders. Figure 1 shows all in-session exclusion criteria and the number of excluded participants. The insulated sequential analysis [34] had three stopping criteria; (1) a one-sided Bayesian Mann–Whitney U test indicated a Bayes Factor ≥10 for or against the prediction that tarantula behavioural approach task approach scores would be higher following the brief reactivation procedure than the extended; (2) less than 50% of participants in the brief condition touched the tarantula after treatment; (3) the maximum sample size of 50 was reached. Data checks were performed after the first 20 participants, then after each 5 (within 1 of equally balanced across groups). For further details, and the results of the interim sequential analyses, see Supplement A. Data collection stopped due to stopping criterion 2) when there were 20 participants in each group. Previously scheduled participants were run and included in the analysis, as their data would still be informative. This resulted in 43 participants, with 23 in the brief condition (20 female, mean_Age_ = 22.04, SD_Age_ = 3.74), and 20 in the extended condition (17 female, mean_Age_ = 21.60, SD_Age_ = 4.60). The University of Amsterdam ethics review board approved all procedures (code 2019-CP-9988), and all participants gave informed consent.

### Materials and measures

#### Questionnaires

Validated questionnaires were used throughout the research (for psychometrics properties see Supplement C).

The Spider Phobia Questionnaire [35] (SPQ; range 0–31) and Fear of Spiders Questionnaire [36] (FSQ; range 0–186) assessed self-reported spider fear, where higher scores indicate more fear. Participants were required to have an SPQ score greater than 17 at pre-screening, in line with previous research [14].

The Patient Health Questionnaire-9 [37] (PHQ-9), State-Trait Anxiety Inventory [38] (STAI), Anxiety Sensitivity Index [39] (ASI), and Structured Clinical Interview for DSM-5-Research Version (SCID-5-RV) were also used. MK supervised training in the SCID-5-RV.

#### Single items

*Subjective Units of Distress* [40] *(SUDS)* – self-reported distress (0 = no distress, 25 = mild distress, 50 = moderate distress, 75 = severe distress, 100 = extreme distress).

*Imagined Behavioural Approach Task (BAT) SUDs* – if participants failed to complete a BAT step, we asked what they thought their SUDs would be if they were to complete it.

*Imagined treatment SUDs* – participants estimated their SUDs if they were to be within touching distance of a tarantula.

*Spider self-efficacy –* confidence ratings that the participant could touch a tarantula at that moment (0–100).

*Treatment credibility ratings* – ratings of the credibility of standard exposure treatment and the experimental treatment (0–100).

#### Reactivation procedure questions

**Fig. 2:**
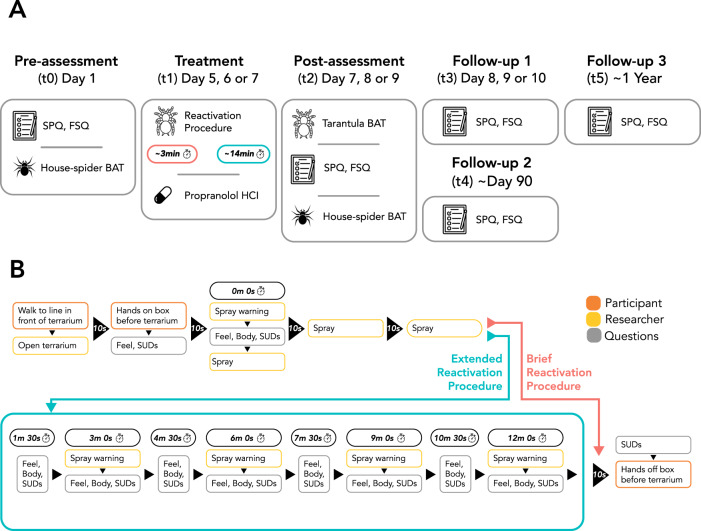
The experimental design. **A** An overview of the key aspects of the experimental procedure. SPQ Spider Phobia Questionnaire; FSQ Fear of Spiders Questionnaire; BAT Behavioural Approach Task. **B** The sequence of events in the reactivation procedures at t1. Stopwatch times indicate time passed since the stopwatch started. If the participant was still responding to the previous questions when the stopwatch reached 1 m 30 s, 4 m 30 s, 7 m 30 s, or 10 m 30 s, these questions were skipped. Feel = participant provided their emotional response; Body = participant provided their physical sensations; SUDs = participant rated their Subjective Units of Distress; Spray warning = researcher warned participant that they would spray the spider with water and emphasised the importance of the participant keeping their hands on the box while this happened; Spray = the researcher sprayed the spider with water until it moved a distance that was at least half the depth of the terrarium.

We asked participants the following to try to facilitate reactivation of the fear memory (Fig. 2B shows timings).

*Pre-treatment spider fear interview* – how they felt when they encountered a spider, and about their first, most recent and worst spider fear experiences. They provided SUDs for each experience.

*Emotional response –* how they felt.

*Physical sensations –* where in their body they felt the reported emotion, or if they reported no emotion, if they were experiencing any physical sensations.

#### Behavioural approach tasks (BATs)

**Fig. 3:**
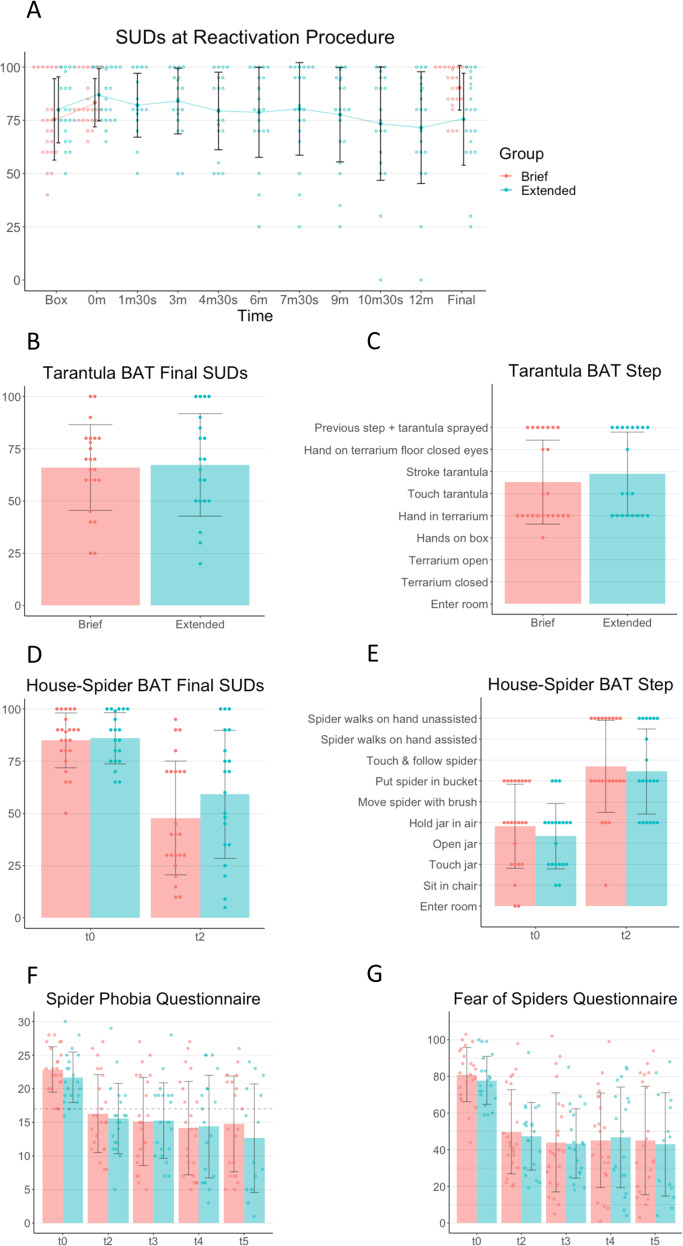
Raw scores and descriptive statistics for the SUDs reported at reactivation procedure and outcome measures. **A** Subjective Units of Distress (SUDs) during the reactivation procedure (t1). Box = participant first placed their hands on the glass box in front of the terrarium; Times = time since participant was first informed that the tarantula would be sprayed with water; Final = final SUDs measurement of the reactivation procedure. **B** Tarantula behavioural approach task (BAT) final SUDs two days after treatment (t2). **C** Tarantula BAT final step two days after treatment (t2). **D** House-spider BAT final SUDs approximately one week before and two days after treatment (t0 and t2). **E** House-spider BAT final step approximately one week before and two days after treatment (t0 and t2). **F** Spider Phobia Questionnaire scores from approximately one week before to one year after treatment (t0 to t5). The horizontal dashed line shows the cut-off for inclusion in the study at pre-screening. **G** Fear of Spiders questionnaire scores from approximately one week before to one year after treatment (t0 to t5). In all panels error bars indicate standard deviations, and circles show individual data points.

The BATs were modified from previous research [14]. Participants approached a spider (a house spider or a tarantula) in steps until they either said “stop” or took more than 3 min to complete the step. Participants reported their SUDs upon completing each step. The final completed step was the BAT score, and tarantula BAT scores were the pre-registered primary dependent variable. Figure 3C, E shows the intermediary steps of the tarantula and house-spider BATs respectively, and Supplement D contains more detailed information.

##### Tarantula BAT

Participants approached an adult grammostola porteri tarantula (leg span approximately 15 cm) in a closed terrarium on a table, with a glass box directly in front of it and a line on the floor 50 cm from the terrarium. Steps ranged from 1 (standing 50 cm from the closed terrarium) to 8 (putting one hand on the ground inside the terrarium and closing their eyes for five seconds while the tarantula was sprayed with water).

##### House-spider BAT

Participants approached a house spider (leg span approximately 4.5 cm) in a glass jar on a table, with a chair 35 cm in front of it. Steps ranged from 1 (sitting 35 cm from the closed jar) to 9 (letting the spider walk on their bare hand without the researcher’s hand around their arm).

#### Physiological measures

Heart rate data were collected continuously during BATs and treatment using a Polar H10 monitor. Analysis of this variable was beyond the scope of the current paper, which focusses on pre-registered variables.

#### Reactivation Procedure

One group underwent a brief (~3 min) and the other an extended (~14 min) memory reactivation (see Fig. 2B for an overview). All reactivation procedures took place in the same room and with the same tarantula as the tarantula BAT. Note that the procedure for the extended group was not only longer, but also included more repetitions of a spray procedure in which the tarantula was provoked to move. The rationale was that each provocation might present a distinct event from which participants could expect a negative outcome, thereby inducing a new prediction error. To enhance fear, the raised glass box was directly before and level with the front opening of the terrarium, so participants felt the tarantula could walk onto their hands when they were on the box.

#### Propranolol

Participants ingested a 40 mg pill of propranolol, with water, within 3 min of the reactivation procedure. Accord Healthcare Ltd. (UK) made the pills, and Huygens Apothecary (NL) provided them. The dosage matches that used in previous research [14].

#### Procedure

See Fig. 2 for an overview of the experimental procedure.

*Researchers and blinding* – graduate research assistants, blind to condition, conducted all screening calls (LB, VM, and MR) and t0 and t2 assessment sessions (LB and VM). The t0 and t2 researcher was consistent within participants. Independent researchers (FR and OdV) performed the stratified randomisation based on t0 scores. AF conducted all treatment sessions (t1) and was only unblinded upon entering the reactivation procedure room to ensure consistency across groups for as long as possible. An independent researcher (JE) conducted the insulated sequential analyses [34], keeping authors and research personnel blind to interim results, beyond whether to continue collecting data.

*Screening* – we assessed eligibility with an online questionnaire and a phone call, which included the SCID-5-RV (Supplement B outlines all exclusion criteria). Participants were informed that the study was comparing the efficacy of two treatments for spider fear in which they would be exposed to spiders and take propranolol HCl.

*Pre-assessment (t0; day 1)* – participants completed the medical screening, then the following questionnaires: SPQ, FSQ, PHQ-9, STAI–t, ASI, and treatment credibility ratings. Participants then performed the house-spider BAT. As in Soeter & Kindt (2015) [14], we did not use the tarantula BAT at baseline to maximise novelty at treatment, as this is thought to be critical to a successful memory reactivation. We excluded participants who touched the spider, in line with previous research [14].

*Treatment (t1; day 5/6/7)* – the STAI-s was followed by a further brief medical screening, including blood pressure and heart rate measurements. We conducted the pre-treatment spider fear interview, to help to reactivate the fear memory, and briefly outlined the treatment mechanism and procedure (identical across groups). Participants rated their Imagined treatment SUDs and Spider self-efficacy, before going to another room for the reactivation procedure determined by their group (see Reactivation Procedure for more details). Afterwards, participants reported their Spider self-efficacy and took 40 mg of propranolol HCl orally. Participants rested with light reading material for 90 min to allow propranolol to reach peak levels, before completing the STAI-s and supplying blood pressure and heart rate measurements.

*Post-assessment (t2; 2 days after t1)* – participants reported their Imagined treatment SUDs and Spider self-efficacy before performing the tarantula BAT. After ten minutes with light reading material, they completed the SPQ and FSQ, waited a further ten minutes, and then performed a house-spider BAT. One participant in the brief condition had this session one week late due to COVID-19 symptoms.

*Follow-up Questionnaires 1 (t3; 3 days after t1), 2 (t4; ~90 days after t1) & 3 (t5; ~1 year after t1)* – participants completed the SPQ and FSQ by email.

#### Statistical analysis

Before analysis, we checked all main dependent variables for outliers that were at least 3 standard deviations from the mean within each group, finding none.﻿ We used Bayesian statistics, and report Bayes Factors (BF) for inference. While the names of these tests match those of their frequentist counterparts, these are calculated differently, to allow explicit evaluation of both null and alternative hypotheses. BF_+_ indicates evidence for the alternative hypothesis (H_+_) over the null hypothesis (H_0_), while BF_0_ is for H_0_ over H_+_. BF_Inclusion_ shows the relative likelihood of observing the data in a model where the specified parameter (i.e., the group or interaction effect) is present versus absent. The Bayesian ordinal logistic regression on the house-spider BAT scores was performed using the R package brms 2.9.0 [41], where we calculated BFs from the sampling distributions by assessing the updating factor for each respective effect size across a null interval of 0 ± 0.1. The priors and further information on how the BFs were calculated can be found in Supplement E, and analysis code used in this model (not pre-registered) is on the Open Science Framework (https://osf.io/2t5nc/). We used JASP for all other tests [42], using the default priors (see Wagenmakers et al. [43] for further details). Bayesian Mann–Whitney U tests used 10,000 samples instead of the default 1000 to enhance the stability of the estimates. If test assumptions were not met, we used non-parametric alternatives. The pre-registered one-sided tests are indicated explicitly.

## Results

### Manipulation checks

Bayesian repeated-measures ANOVAs found that resting heart rate, and systolic and diastolic blood pressure dropped in both groups from before to 90 min after propranolol HCl administration (all BF_Inclusion_Time_ ≥ 474.26), without evidence that these changes were affected by group (all BF_Inclusion_Group*Time_ ≤ 2.23). The effect sizes of the reduction in blood pressure and heart rate from the start to the end of the treatment session (t1) were comparable to those in previous research [14], indicating that propranolol was physiologically active (η^2^_Systolic_Blood_Pressure_ = 0.431, η^2^_Diastolic_Blood_Pressure_ = 0.346, η^2^_Heart_Rate_ = 0.797; for descriptive statistics see Supplement F).

Bayesian independent samples *t*-tests suggested equivalence of the groups across the first two SUDs ratings of the treatment, indicating that the experience was similar until the treatment protocols diverged and equivalent levels of baseline tarantula fear (BF_+_s ≤ 0.458; see Fig. 3A for the SUDs reported during treatment).

### Baseline characteristics

The groups did not differ on baseline characteristics (BF_+_ < 1; see Table 1), except for some evidence for a group difference on clinical status (BF_+_ = 1.44).

**Table 1:** Mean scores [SD] by group for baseline characteristics and additional single-item questions.

	Time	Condition		BF_+_
		Brief (*n* = 23)	Extended (*n* = 20)	
*Baseline measures*				
Age (years)	t0	22.0 [3.7]	21.6 [4.6]	0.316
Anxiety Sensitivity Index	t0	20.2 [11.2]	19.0 [11.3]	0.316
Clinical status	Screening call	61% clinical	35% clinical	1.44
		(*n* = 14)	(*n* = 7)	
Fear of Spiders Questionnaire	t0	81.0 [14.8]	77.9 [13.1]	0.373
House-spider BAT step	t0	3.8 [2.0]	3.4 [1.6]	0.390
House-spider BAT final step SUDs	t0	85.0 [13.1]	86.0 [12.3]	0.309
Imagined BAT SUDs	t0	97.8 [5.1]	97.7 [5.3]	0.363
Patient Health Questionnaire-9	t0	4.7 [3.8]	3.4 [3.2]	0.537
Sex	t0	87% female	85% female	0.499
Spider Phobia Questionnaire	t0	22.9 [3.4]	21.7 [3.8]	0.478
State-Trait Anxiety Inventory – trait subscale	t0	40.8 [10.0]	39.1 [11.0]	0.338
Experimental treatment credibility	t0	61.7 [19.9]	64.9 [19.8]	0.334
Exposure treatment credibility	t0	62.1 [22.1]	60.6 [26.5]	0.306
*Additional Single Items*				
Self-efficacy score	t1-pre	3.6 [6.5]	3.5 [7.3]	0.386
	t1-post	12.9 [15.3]	29.0 [28.3]	0.641
	t2	14.4 [15.5]	18.1 [19.7]	0.363
Imagined treatment SUDs	t1-pre	88.9 [15.5]	90.5 [15.0]	0.330
	t2	70.2 [19.3]	69.5 [21.7]	0.317

Bayes Factors (BFs) are from Bayesian two-tailed independent samples t-tests comparing the brief and extended condition, except for the house-spider BAT step, imagined BAT SUDs, self-efficacy scores, and imagined treatment SUDs, which were tested with Bayesian Mann–Whitney U tests, and sex and clinical status, which were tested with a Bayesian chi-square. *BAT* Behavioural Approach Task; *SUDs* Subjective Units of Distress; t0 = Pre-assessment; t1-pre = Before the treatment in the treatment session; t1-post = Directly after treatment in the treatment session; t2 = Before the tarantula BAT at post-assessment.

### Confirmatory analyses

In the primary confirmatory analysis, a one-sided Bayesian Mann–Whitney U test found that the two groups did not differ in approach scores in the tarantula BAT, against the hypothesis that the brief group would approach the tarantula more (BF_0_ = 4.80; Fig. 3B). A one-sided Bayesian independent samples *t*-test also found some evidence against the prediction that the brief group would have lower distress at the final completed step in the tarantula BAT (BF_0_ = 2.91; Fig. 3C). Overall, this indicates that the two groups did not differ in their tarantula approach or distress upon doing so.

A Bayesian ordinal logistic regression found strong evidence that participants were more able to approach the house spider after the treatment (0% approached less; BF_+_ = ∞; Fig. 3D), without evidence for the predicted group-by-time interaction (BF_0_ = 1.71) nor a group effect (BF_0_ = 2.40). Bayesian repeated-measures ANOVAs assessed the changes from before to after treatment on all other measures. We found strong evidence for a decline in SUDs at the final completed step of the house-spider BAT from t0 to t2 (BF_Inclusion_Time_ = 7.73e^7^, BF_Exclusion_Group_ = 2.10, BF_Exclusion_Time*Group_ = 1.47; Fig. 3E).

Participants’ SPQ scores decreased strongly between pre-assessment and one year after treatment (t0, t2, t3, t4, and t5; 7% increased score by 1 at the final completed questionnaire; BF_Inclusion_Time_ = ∞; Fig. 3F), with evidence against the hypothesis that this reduction was affected by treatment duration (BF_Exclusion_Condition*Time_ = 9.51), such that both groups maintained mean SPQ scores that were below the inclusion threshold of 17 from two days after treatment up until the final measurement one year later. A similar pattern was observed for the FSQ scores (7% increased scores by final completed questionnaire, ranging from 3 to 11; BF_Inclusion_Time_ = 3.00e^15^, BF_Exclusion_Condition*Time_ = 16.28; Fig. 3G). All participants improved on at least one outcome measure.

As there was no evidence for an effect of group on these scores, we collapsed the two groups in the follow-up Bayesian paired sample *t*-tests. Both SPQ and FSQ scores abruptly decreased from t0 to t2 (BF_+_ = 4.39e^7^ and BF_+_ = 3.43e^9^ respectively). A suggested further reduction in FSQ scores from t2 to t3 (BF_+_ = 3.184), which was maintained from t3 to t4 (BF_0_ = 5.13) and t4 to t5 (BF_0_ = 5.51), did not translate to a reduction from t2 to t5 on the FSQ (BF_0_ = 2.25). Contrastingly, on the SPQ, while there was no compelling evidence that scores dropped between t2 and t3 (BF_+_ = 1.90), t3 and t4 (BF_0_ = 1.70), nor t4 and t5 (BF_0_ = 5.23), there was a decrease from t2 to t5 (BF_+_ = 3.11). Overall, this indicates that scores on both spider fear questionnaires strongly reduced from one week before treatment to two days after treatment, where FSQ scores then remained broadly stable, while SPQ scores declined further in the following year.

## Discussion

Unexpectedly, we found no effect of reactivation procedure duration on treatment outcomes. Instead, both groups showed a substantial reduction in spider fear across both behavioural and self-report measures after treatment, without relapsing one year later. While the results suggest a large and enduring treatment effect, the mechanisms are unclear, as there are neither differences between the groups, nor placebo control groups to compare to. A direct comparison with the findings of Soeter and Kindt [14] may provide some insight as the research that is closest to the current study, although participants were more fearful in the current research (reflected in higher baseline spider fear on the Spider Phobia Questionnaire and house-spider behavioural approach task). In our study, treatment outcomes were consistently better than in the control groups in Soeter and Kindt’s study, and roughly equivalent to those of their treatment group, except for tarantula approach behaviour (the primary outcome variable) which was lower in the current study than in their treatment group. Together with the indications that propranolol was physiologically active, this leaves the following scenarios to explain the current results: (A) neither the brief nor the extended reactivation procedures triggered reconsolidation, (B) in one group reconsolidation was triggered, and an alternative process caused an equally large treatment effect in the other group, (C) reconsolidation was triggered by both the brief and extended reactivation procedures.

### Scenario A: Neither the brief nor extended reactivation procedures triggered reconsolidation

Beyond the lower levels of tarantula approach behaviour after treatment, the most compelling evidence for this scenario is that the self-reported spider fear declined sooner than expected. In Soeter and Kindt’s study, spider fear questionnaire scores remained high one week after the tarantula behavioural approach task, only decreasing at the next measurement point three months later. Similarly, in fear-conditioning research, disrupting reconsolidation affects behavioural and cognitive fear expression differently, reducing the behavioural fear response (i.e., the fear-potentiated startle) immediately after a night of sleep, while cognitive expression of fear (i.e., shock expectancy ratings) remains unchanged [26]. Contrastingly, we found that behavioural and cognitive changes were aligned, as self-reported spider-fear scores reduced in the same session as the behavioural approach tasks. This may indicate that alternative mechanisms are at play.

If reconsolidation is not the mechanism, we must consider which processes may have caused the observed reduction in spider fear, which compares to that seen after 2.5 hr of in vivo spider exposure [31]. We neither administered placebos nor told participants we would, to motivate participants to “open up” to the reactivation procedure experience, believing that suppressing emotions may inhibit reconsolidation. The extended condition served as the control group, as supported by the ineffectiveness of the extended reactivation procedure during piloting. While the propranolol may have blocked the consolidation of extinction learning [24], the certainty of taking an active drug may have enabled participants to immerse and push themselves in the reactivation procedures more than they typically would in standard exposure treatments, enhancing other effects, such as changes in self-efficacy [44]. Additionally, as the story provided about the effects of a placebo, when the placebo is given in combination with exposure, affects the long-term impact of that exposure [45], perhaps the interpretation of our intervention enhanced treatment effects (i.e., a placebo effect), rather than the content of the reactivation procedure. This appraisal of the intervention would not necessarily vary with the duration of the exposure.

### Scenario B: In one group reconsolidation was triggered, and an alternative process caused an equally large treatment effect in the other group

As we cannot directly observe the mechanisms at play in the two groups, different processes may have occurred across the groups, with equivalent effects on our outcome measures. Given the brevity of the reactivation procedure employed by Soeter and Kindt, it seems more plausible that reconsolidation was triggered by the brief rather than the extended reactivation procedure. While the propranolol would have inhibited extinction learning, the processes described in Scenario A (i.e., certainty of taking an active pill leading to participants pushing themselves more, and their appraisal of the treatment reducing relapse) may have enhanced the effects of the extended reactivation procedure. Some evidence for this hypothesis could be that the extended reactivation procedure increased spider self-efficacy more than the brief reactivation procedure directly after treatment (see Table 1).

This scenario, however, does not explain the discrepancy between the current study’s brief reactivation procedure condition and Soeter and Kindt’s treatment group regarding treatment effects on tarantula approach behaviour and the timeline of self-reported spider fear.

### Scenario C: Reconsolidation was triggered by both the brief and extended reactivation procedures

While there are differences in the results of our treatment when compared to that of Soeter and Kindt on some outcomes (i.e., lower tarantula approach and earlier self-reported spider fear change), generally, the fear reduction is very similar in size and stability. It may be that despite efforts to induce more prediction error in the extended group (e.g., by increasing the duration of time spent in proximity to the spider, and the number of provocation events by means of sprays), both the brief and extended reactivation procedures induced the same number of prediction error events. For example, if participants only expected a negative outcome each time they placed their hands on the box, then the number of prediction error events would have been the same across both conditions as the protocols did not differ in this regard. Alternatively, perhaps the extended reactivation procedure was insufficiently long to trigger the limbo state or extinction learning. This may seem unlikely, considering that fear-conditioning research found mere additional seconds of reactivation procedure to be sufficient to inhibit reconsolidation [7], yet recent evidence suggests that this window may widen as the strength of the memory increases. As mentioned previously, in crabs, both small and medium numbers of CS-alone presentations triggered reconsolidation for a stronger memory, while only the small number triggered reconsolidation of the weaker memory [5]. Since clinical fears involve highly emotional acquisition experiences, which can compound across multiple timepoints over many years, the boundaries of duration that can trigger reconsolidation may be significantly wider in clinical practice than the fear-conditioning research suggests.

It is also possible that the reactivation procedure was only suitable for some participants, resulting in a combination of scenarios A and C (i.e., reconsolidation occurred in some individuals but not others, in both conditions). As we systematically varied the reactivation procedure, it had to be highly standardised. To try to improve the chances that this standardised reactivation procedure would be suitable we used strict inclusion criteria, for example, increasing the chances that a tarantula would be the correct spider to maximally reactivate their fear. Despite these efforts, at baseline there was still a higher proportion of included participants with a clinical status in the brief condition. This could have obscured group differences in treatment effect. For the excluded individuals, some may still have been suitable for reconsolidation interventions in clinical practice, as in the clinic, interventions can be tailored to an individual, whereas we tailored the sample to our intervention. While we do not have an independent marker of reconsolidation, we cannot determine on an individual level whether reconsolidation is responsible for changes in fear expression following treatment.

## Conclusions

Contrary to expectations, we found that the length of the reactivation procedure did not affect the outcome of a reconsolidation intervention: both the brief and extended reactivation procedures resulted in an abrupt reduction in spider fear. Without a non-invasive marker for prediction error or reconsolidation, it remains unclear why we did not find the predicted effect. While this translation of the laboratory studies was based on just one clinical interpretation of prediction error, many other possibilities remain. Future research should explore other avenues, such as repeatedly approaching the feared stimulus (e.g., placing hands on the box in front of the tarantula), or leaving and re-entering the room containing the feared stimulus, to ensure that there are concrete distinct episodes of exposure. Our findings also demonstrate the importance of including placebo controls in such research to clarify the mechanisms of any treatment effects. Regardless, the marked improvement that followed a single brief exposure session combined with an amnesic agent gives a tentative promising message for the future of reconsolidation interventions. While approximately half the participants had a clinical spider phobia, all participants who completed the treatment improved on at least one outcome measure, and none experienced a significant worsening of spider fear. Our findings may indicate that the boundary conditions for reconsolidation are wider in naturalistic fears than the fear-conditioning literature indicates, or that treatments involving a combination of a placebo and exposure can yield large and stable effects via alternative mechanisms. Further, we tested an approach to uncover the boundary conditions of reconsolidation in naturalistic fears with unknown learning histories. Such translational efforts are critical to making reliable reconsolidation interventions a viable clinical reality, as spider fear can act as an intermediary translational step between fears conditioned in the laboratory and more complex emotional memory disorders.

## Supplementary information

Supplement
