# Supplementary material for: Over the Edge: Extending the duration of a reconsolidation intervention for spider fear
Source: Transl Psychiatry. 2022 Jun 23;12:261. doi: 10.1038/s41398-022-02020-x (PMC9219370; doi:10.1038/s41398-022-02020-x)
Supplement: Supplementary file 1 — Supplement [file 41398_2022_2020_MOESM1_ESM.docx]

**Supplementary Materials**

Supplement A: The Sequential Analysis

As the pseudo-randomisation occurred before treatment, and there were high exclusion levels at treatment, exact matching of group sizes was not always possible. Interim analyses were performed on the number of participants that was prespecified for that analysis, regardless of whether more participant data had been collected (i.e., if there were too many participants in one condition for the check, the extra participants were not included in that check).

The sequential analysis used a Bayesian Mann-Whitney U test conducted in JASP, comparing the brief and extended groups on their tarantula behavioural approach task scores (the primary dependent variable). The default settings were used, other than that the number of samples was increased from 1,000 to 10,000, and that a one-sided test was used, with the prediction that the brief group would have higher approach scores than the extended group. To further enhance the stability of the estimates, this analysis was conducted 10 times per data check, and the median BF was compared with the cut-off value. The results of the intermediary analyses are in Supplementary Table 1.

| **Supplementary Table 1.** Descriptive statistics and results of the interim analyses. | | | | |
| --- | --- | --- | --- | --- |
| *Data check* | Group | Number of participants | Mean tarantula BAT score [SD] | Median BF_0_ |
| 1 | Brief | 10 | 6.0 [1.9] | 1.91 |
|  | Extended | 10 | 5.7 [1.8] |  |
| 2 | Brief | 13 | 5.8 [1.9] | 2.60 |
|  | Extended | 12 | 5.8 [1.9] |  |
| 3 | Brief | 15 | 5.6 [1.8] | 3.63 |
|  | Extended | 15 | 5.9 [1.9] |  |
| 4 | Brief | 18 | 5.6 [1.8] | 4.09 |
|  | Extended | 17 | 5.9 [1.9] |  |

Median Bayes Factors (BF_0_s) are calculated from 10 one-tailed independent samples Mann-Whitney U tests comparing the tarantula BAT scores between the brief and extended groups and show the evidence against the prediction that the brief group would have higher scores than the extended group. BAT = Behavioural Approach Task.

Supplement B: Exclusion Criteria

As participants were required to take propranolol HCl, there were strict medical inclusion criteria. To ensure that the medical information had not changed during the study, this information was collected at multiple timepoints; in the screening questionnaire, on the follow-up phone call, at pre-assessment (t0), and at treatment (t1). Blood pressure and heart rate measurements were only collected at t0 and t1 as these were in-person sessions. Specifically, the following were the medical exclusion criteria:

- personal history of heart problems, vascular diseases, or an irregular heartbeat
- current asthma and other lung disorders that were not adequately regulated with medication
- personal history of allergic reactions to propranolol
- planning to become or currently being pregnant or breastfeeding
- systolic blood pressure below 100 or a diastolic blood pressure below 60
- resting heart rate below 60, or a resting heart rate below 50 if they exercised for more than 7 hours a week
- heart rate did not rise from the resting heart rate after two minutes of light exercise (walking up and down two steps repeatedly)
- taking any of the following medications: medication that works on the heart, blood pressure lowering agents, ergotamine preparations, insulin, antacids, anti-inflammatory painkillers, antidepressants, antipsychotics, anti-anxiety medication, asthma medication, medication against dizziness, tuberculosis or psoriasis, or any other medicine that interacts with propranolol

There were also non-medical inclusion criteria. Some unanticipated exclusions were also applied, which were not included in the pre-registration. These additional criteria are marked below with an asterisk (*), and the rationale for the exclusion is given. Decisions about these criteria were made blind to a participant’s post-assessment session (t2), so they would be unaffected by the outcome of the intervention. Specifically, the following exclusion criteria applied:

**Online screener**

- not comfortable conducting the research in English
- not aged between 18 and 50
- a Spider Phobia Questionnaire score below 18
- more afraid of house spiders than tarantulas
- more disgusted by spiders than afraid of them
- a Spider self-efficacy score of above 25. In the screener this was assessed by asking participants to how confident that they were that they could touch a tarantula “right now” on a visual analogue scale of 0 (“I definitely could not do it”) to 100 (“I definitely could do it”)
- a Patient Health Questionnaire-9 score of 15 or higher, as this indicates moderately severe depressive symptoms
- used a beta-blocker more than twice in the past two-years
- a current mental health diagnosis, other than ADHD or ADD
- a previous diagnosis of psychoticism
- previous therapy for their spider fear that included exposure to a tarantula
- previous participation in any research relating to their fear of spiders where they encountered a spider in person, or where they entered the room in which the memory reactivation and Behavioural Approach Tasks (BATs) in this research took place

**Pre-assessment (t0)**

- touched the spider in the house-spider BAT – otherwise they were deemed insufficiently fearful
- * were too fearful to enter the BAT room. This was selected on the rationale that if the participant could not enter the spider room for the BAT, they would not be able to complete the treatment procedure.
- * experimenter unintentionally violated the house-spider BAT protocol, as the participant was allowed to try the step for longer than 3 minutes. We excluded this participant, as the extra time led to additional exposure occurring for that participant during the BAT, which may have affected their future data.
- * being unable to return to the laboratory after it was closed due to the COVID-19 crisis. Some participants had already attended their pre-assessment session. Participants who were able to return after the laboratory had reopened started the study again and completed the t0 session again, to ensure that the elapsed time had not affected their scores. Some participants were unable to return to the laboratory for practical reasons.

**Treatment (t1)**

Please note – the exclusion criteria at treatment were only applicable to parts of the reactivation procedure that were the same across groups, to avoid group-specific exclusions. The exclusion criteria were:

- low distress at the onset of the reactivation procedure, defined by not reporting at least one Subjective Units of Distress (SUD) rating of 65 or higher in the first two SUDs of the reactivation procedure
- unable to place their hands on the box in front of the terrarium within the first two minutes of the reactivation procedure, or to keep their hands down for two sprays within 3 minutes of being warned that the spider will be sprayed
- a pre-treatment Spider self-efficacy above 25. This was assessed verbally (see Single Items in the Materials and Measures section)
- reporting more disgust than fear upon encountering a spider in the spider fear interview
- terminating the reactivation procedure due to high distress
- * highly unusual tarantula behaviour during the treatment. This criterion was met if the tarantula exited the terrarium during the reactivation procedure. We decided to exclude these participants as their experience during the reactivation procedure was very different to that of other participants.

Supplement C: Description and Psychometric Properties of the Questionnaires

**Anxiety Sensitivity Index** (ASI; Peterson & Reiss, 1992) – a 16-item self-report questionnaire, assessing beliefs about the consequences of anxiety symptoms, with good reliability (α = 0.88; Peterson & Heilbronner, 1987), and validity, where ASI scores predicted panic attacks (Weems, Hayward, Killen, & Taylor, 2002).

**Fear of Spiders Questionnaire** (FSQ; Szymanski & O'Donohue, 1995) – an 18-item self-report questionnaire using a 7-point Likert response format, where a higher score indicates higher spider fear with good reliability (α = .95-.97), and validity, correlating with behaviour in a spider approach task and being able to differentiate between individuals with clinical spider phobia and non-phobic controls (Muris, & Merckelbach, 1996).

**Patient Health Questionnaire 9** (PHQ-9; Kroenke, Spitzer, & Williams, 2001) – a 9-item self-report questionnaire assessing depression, with good reliability (α = .86-.89), and validity where a comparison with clinician diagnoses of depression showed 88% in both specificity and sensitivity (Kroenke et al., 2001).

**State-Trait Anxiety Inventory** (STAI; Spielberger, Gorsuch & Lushene, 1970) –– a 40-item self-report questionnaire, assessing state and trait anxiety, with good reliability (α = 0.86-0.95; Spielberger et al., 1970), and validity with a correlation with the Scheier's Anxiety Scale Questionnaire of .85 (Cattell & Sheier, 1963).

**Spider Phobia Questionnaire** (SPQ; Klorman, Weerts, Hastings, Melamed, & Lang, 1974) – a 31-item self-report questionnaire with a true-false response format, where higher scores indicate higher spider fear. It has good reliability (test-retest correlation = .94), internal consistency (α = .91), and validity, as it can differentiate between phobic and non-phobic controls (Muris, & Merckelbach, 1996).

Supplement D: The Behavioural Approach Tasks (BATs)

Before all BATs, participants were instructed that the purpose of the task was to assess their fear, that they could terminate the task at any time by saying “stop”, and that there was no need to push themselves beyond their limit. If participants took longer than 3 minutes to complete a step, the BAT was terminated, however participants were not informed about the time limit. Each step instruction was given separately, in the specified order. Participants could not move on to the following step until the current step was complete (one exclusion to this rule is noted below). See Supplementary Table 2 for the steps of both the house-spider and tarantula BATs.

*House-spider BAT*

This was modified from Soeter and Kindt’s baby tarantula BAT (2015) to add an optional step where the experimenter placed their hand around the participant’s upper arm while the spider walked on the participant’s hand. This was due to our experience that participants would avoid the spider walking on their hands as they feared it would run up their arm. Participants could also choose to progress directly to step 9 and skip step 8. See Table 1 for the steps of the house-spider BAT.

*Tarantula BAT*

We modified this from the adult tarantula BAT used in Soeter and Kindt’s study (2015) to include additional steps, where the final step was touching the tarantula (here Step 5 of 8). This was due to a low variation in the outcomes in Soeter and Kindt, where all participants in the active treatment condition completed the final step of the adult tarantula BAT.

| **Supplementary Table 2.** The steps of the spider behavioural approach tasks. |
| --- |
| **Tarantula Behavioural Approach Task** |
| 1. Stand 50cm from the closed terrarium. |
| 1. Stand 50cm from the open terrarium. |
| 1. Place both hands flat down on the glass box directly in front of the open terrarium. |
| 1. Put one hand in the middle of the air in the terrarium space. |
| 1. Touch the tarantula on the back. |
| 1. Stroke the spider for five seconds. |
| 1. Put one hand on the ground inside the terrarium and close eyes for five seconds. |
| 1. Put one hand on the ground inside the terrarium and close eyes for five seconds while the tarantula is sprayed with water. |
| **House-Spider Behavioural Approach Task** |
| 1. Sit 35cm from the jar containing a house spider. |
| 1. Hold the closed jar for 10 seconds. |
| 1. Open the jar. |
| 1. Hold the open jar in the air for 10 seconds. |
| 1. Direct the spider’s movement with a paintbrush for 10 seconds. |
| 1. Put the spider from the jar into a bucket. |
| 1. Touch the spider with their index finger and follow it for 10 seconds. |
| 1. (Optional) Let the spider walk on their bare hand with the researcher’s hand around the top of their arm. |
| 1. Let the spider walk on their bare hand without the researcher’s hand around their arm. |

Supplement E: Further information about the Bayesian Ordinal Logistic Regression

We set the priors to be broadly uninformative to reduce bias, but still realistic, as follows.

Condition – student_t(nu = 5, m = 0 , sd = 1.25)

Time - student_t(nu = 5, m = 0 , sd = 1.25)

Condition*Time - student_t(nu = 5, m = 0 , sd = 0.625)

Intercept - student_t(nu = 5, m = 0 , sd = 1.5)

Standard Deviation - student_t(nu = 5, m = 0 , sd = 1.25)

Using the R-package brms, we created both a prior-only model, and a model that took both the prior and the observed data into account. For each effect of interest, we then extracted posterior samples from both distributions and compared the density of these posterior distributions over our null interval (±.1), to detect whether the model assigned a higher likelihood to the effect falling within this null interval after observing the data. The full analysis code can be found on the Open Science Framework (<https://osf.io/2t5nc/>).

Supplement F: Descriptive Statistics of Blood Pressure and Heart-rate Measures

See Supplementary Table 3 for the descriptive statistics of the blood pressure and heart rate measurements obtained before and 90 minutes after propranolol HCl was administered.

| **Supplementary Table 3.** Mean scores [SD] by group for physiological measures collected at t1. Heart rate is shown in beats per minute. | | | | |
| --- | --- | --- | --- | --- |
| Time | Group | Systolic Blood Pressure | Diastolic Blood Pressure | Heart rate |
| Before propranolol | Brief | 114.50 [9.42] | 72.57 [7.26] | 74.94 [10.43] |
|  | Extended | 114.08 [7.86] | 69.93 [5.17] | 72.00 [11.02] |
| 90 mins after propranolol | Brief | 110.50 [8.12] | 68.67 [7.56] | 54.76 [5.51] |
|  | Extended | 105.58 [5.90] | 67.00 [5.68] | 53.58 [9.30] |
